# Associations between patient-reported late effects and systemic cytokines in long-term survivors of head and neck cancer treated with radiotherapy

**DOI:** 10.1007/s11764-022-01273-1

**Published:** 2022-11-09

**Authors:** T. T. M. Huynh, H. C. D. Aass, R. S. Falk, G. L. Astrup, Å. Helland, T. Bjøro, K. Bjordal, E. Dale, T. P. Hellebust, B. B. Herlofson, E. Malinen, C. E. Kiserud, T. Osnes, C. D. Amdal

**Affiliations:** 1 Faculty of Medicine, University of Oslo, Oslo, Norway; 2 Department of Oncology, Oslo University Hospital, Post Box 4950, NO-0424 NydalenOslo, Norway; 3 Department of Medical Biochemistry, Oslo University Hospital, Oslo, Norway; 4 Research Support Services, Oslo University Hospital, Oslo, Norway; 5 Department of Physics, University of Oslo, Oslo, Norway; 6 Department of Medical Physics, Oslo University Hospital, Oslo, Norway; 7 Faculty of Dentistry, University of Oslo, Oslo, Norway; 8 Department of Otorhinolaryngology, Head and Neck Surgery, Oslo University Hospital, Oslo, Norway

**Keywords:** Head and neck cancer survivors, Radiotherapy, Late effects, Patient-reported outcomes, Health-related quality of life, Pro-inflammatory cytokines

## Abstract

**Purpose:**

Head and neck cancer (HNC) treatment may lead to late effects and impaired health-related quality of life of survivors. Knowledge on long-term late effects after radiotherapy (RT) and potential underlying biological mechanisms is lacking. We assessed the prevalence of xerostomia, dysphagia, and chronic fatigue (CF) in HNC survivors ≥ 5 years post-RT, and examined associations between pro-inflammatory cytokines and late effects.

**Methods:**

In a cross-sectional study, 263 HNC survivors treated between 2007 and 2013 were enrolled. They completed validated questionnaires assessing xerostomia and dysphagia (the EORTC QLQ-H&N35), and CF (the Fatigue Questionnaire), and underwent blood sampling and clinical examination. Pro-inflammatory cytokines were analyzed in 262 survivors and 100 healthy age- and gender-matched controls.

**Results:**

Median time since treatment was 8.5 years. The proportions of survivors reporting xerostomia, dysphagia, and CF were 58%, 31%, and 33%, respectively, with a preponderance of females. We found no significant associations between IL-6, IL-8, IP-10, TARC, TNF, or ENA-78 and the three late effects. The odds of having elevated levels of IL-6 and IP-10 were significantly higher in the survivors compared to the controls.

**Conclusions:**

More than one-third of long-term HNC survivors experienced xerostomia, dysphagia, and CF. Persistent inflammation, with elevated systemic cytokines, was not associated with these late effects, although HNC survivors had higher levels of some cytokines than the controls.

**Implications for Cancer Survivors:**

This study provides new knowledge on late effects that can serve as grounds for informing patients with HNC about risk of late effects more than 5 years after RT.

**Supplementary Information:**

The online version contains supplementary material available at 10.1007/s11764-022-01273-1.

## Introduction

The prevalence of long-term survivors after treatment of head and neck cancer (HNC) is increasing [1, 2]. Consequently, a substantial proportion may live with late effects as the head and neck area encompasses vital and susceptible structures subjected to treatment-related harm. Lasting functional, physiological, and esthetic consequences commonly affect health-related quality of life (HRQoL) [3–6]. Knowledge of underlying biological mechanisms of cancer- and treatment-related late effects is lacking. A more comprehensive understanding of these mechanisms is crucial to identify individuals at risk of developing severe late effects, thereby enabling optimized preventive measures, follow-up, and supportive care.

In HNC, late effects are usually defined as disease- or treatment-related side effects occurring or persisting more than 3 months post-treatment [7, 8]. Most late effects are subjective and the patients themselves should be the primary source of information. Such data can be collected successfully using validated patient-reported outcome measures (PROMs) [9–11]. Current knowledge on late effects in HNC patients is mostly derived from the early years following treatment. Pain, swallowing difficulties, problems with dry mouth and sticky saliva, hoarseness, edema, and fatigue are frequently reported up to 5 years after treatment [12, 13]. Literature concerning late effects in survivors beyond this time span is scant [7, 14], specially related to patients receiving modern radiotherapy (RT). Hence, more knowledge on the prevalence and possible biological mechanisms for persistent xerostomia, dysphagia, and chronic fatigue (CF) more than 5 years post-treatment is needed and will be addressed in this study. Approximately 15 years ago, two major changes in treatment of HNC were introduced that might influence late effects: intensity-modulated radiotherapy (IMRT) and concomitant chemotherapy [15–18]. IMRT reduces the dose to normal tissue compared to 3D-conformal RT [15], but still the prevalence of xerostomia in HNC survivors is reported to be up to 40% and 30% at 12 months and 24 months post-treatment, respectively [19, 20]. To date, the literature has not uniformly elucidated the prevalence of dysphagia in HNC survivors as studies have used different measurement methods and dysphagia-related endpoints [6, 21]. Fatigue is a distressing and subjective sense of physical, emotional, and/or cognitive tiredness [22–24]. The most established definition of CF is fatigue that lasts 6 months or longer [25]. In general, cancer patients report increased fatigue in the first year, and 20–25% report CF several years following treatment, potentially deteriorating HRQoL [12, 22, 26].

There is growing evidence for the role of inflammation in the progression of cancer and treatment-related side effects [24, 27, 28]. Pro-inflammatory cytokines have been shown to be activated by malignant tumors and cancer treatment [3], but an association with late effects in HNC survivors has not been described [24]. Cytokines such as interleukin (IL)-1 beta (IL-1β), IL-6, IL-8, and tumor necrosis factor (TNF) have been linked to oral mucositis, and possibly xerostomia, during RT, manifested by elevated levels in saliva immediately after treatment in HNC patients [29, 30]. Chronic inflammation may further cause fibrosis, one of the key pathological features in radiation-induced late effects such as dysphagia [4, 31]. Transforming growth factor beta (TGF-β) and TNF are involved in the formation of fibrosis in irradiated tissue, predominantly found in lung cancer studies [4, 32–35]. The etiology and pathophysiology of CF remain unclear, but significantly higher serum levels of pro-inflammatory cytokines have been measured in individuals with cancer-related fatigue compared to non-fatigued survivors or healthy controls [26–28, 36].

The main objective of the present study was to provide knowledge on late effects after RT in HNC survivors and associations with systemic inflammatory markers. We investigated the (1) prevalence of patient-reported xerostomia, dysphagia, and CF in long-term HNC survivors, (2) systemic cytokine levels in HNC survivors and controls, and (3) associations between cytokines and late effects in long-term HNC survivors.

## Materials and methods

### Data collection and patients

This cross-sectional HNC survivorship study was conducted at Oslo University Hospital (OUH), Norway, over a 2-year period from October 2018. The study was linked to an international multi-center study by the European Organisation for Research and Treatment of Cancer (EORTC) on late toxicity and quality of life in HNC survivors [37]. Two user representatives, both HNC survivors, were involved as project partners from the start. The Regional Committees for Medical and Health Research Ethics (reference number 2018/1005), the local protocol committee, and the OUH privacy office approved the study.

Eligible survivors were aged ≥ 18 years at survey and had received RT for HNC at OUH in the period 2007–2013. They had ability to understand and respond to PROMs, and to attend a 1-day visit at OUH. The study team identified the HNC survivors in the hospital diagnosis register. An invitation letter with study information and consent form was sent by mail to eligible candidates. A subgroup of participants was also invited to participate in the international EORTC multi-center study. HNC survivors who agreed to participate provided written informed consents before inclusion.

Participants completed a set of validated PROMs prior to the visit, including the EORTC core questionnaire (QLQ-C30) [38], the HNC specific module EORTC QLQ-H&N35 [39], and the Fatigue Questionnaire (FQ) [40]. During the visit at the hospital, the study coordinator checked the completed questionnaires together with the participant and addressed any questions in order to minimize missing items. On arrival, the laboratory collected fasting blood samples and the participant met with a clinician at the oncology outpatient department. The appointment included clinical examination of the head and neck area and assessment of weight, body mass index (BMI), blood pressure, and WHO performance status (PS). The medical history and treatment details were extracted from the medical record and radiation registry system. Comorbidities were scored according to the Charlson Comorbidity Index [41].

### Treatment

Treatment was applied according to the Danish Head and Neck Study Group (DAHANCA) guidelines [42]. The standard primary RT regimen for patients with curative intention was 2 Gy per fraction, 5 to 6 days a week, up to a total dose of 70 Gy to the tumor and lymph node metastases, and 46 Gy to the elective neck regions with concomitant nimorazole, a hypoxic radiosensitizer [43–45]. Patients < 70 years with stage III and IV disease also received concomitant weekly cisplatin 40 mg/m^2^ [46, 47]. In the postoperative setting, patients received a total dose of 50–66 Gy, 2 Gy per fraction, to the tumor bed(s), and 46 Gy to the elective neck regions, 5 days a week, with or without weekly cisplatin.

### PROMs and definition of selected late effects

The EORTC QLQ-H&N35 was used to measure patient-reported xerostomia and dysphagia as endpoints in this study. It consists of 35 questions arranged in seven scales (pain, swallowing, senses, speech, social eating, social contact, and sexuality) and six single items (teeth, opening mouth, dry mouth, sticky saliva, coughing, and felt ill). The items had responses on Likert scales ranging from 1 = “not at all” to 4 = “very much” [38, 39]. The scores from scales and single items were linearly transformed to scores ranging from 0 to 100. High scores on the symptom scales indicated more severe symptoms [48]. In this study, dysphagia was defined as a score of ≥ 25 on the dysphagia scale (q5–q8) and xerostomia as ≥ 66 on the dry mouth single item (q11).

The FQ consists of 11 items related to mental and physical fatigue. Each item was answered on a 4-point Likert scale. The dichotomous variant of the scoring procedure was used: “better than usual” and “no more than usual” scored as 0 and “worse than usual” and “much more than usual” scored as 1. Accordingly, summation gave a total score of maximum 11. Two additional items evaluated the duration and extent of fatigue symptoms. CF was defined as a dichotomized score of ≥ 4 lasting 6 months or longer [49].

### Sample collection

Serum was collected using standard procedure (Supplementary S1, Online Resource 1) from all study participants and 100 blood donors. The healthy controls were frequency-matched with the study group by age and gender (Supplementary S1, Online Resource 1), and consisted of 30 females (median age 62 years, range 47–68) and 70 males (median age 62 years, range 43–68).

### Quantification of cytokines

Cytokine analyses were performed using a Luminex IS 200 (Bio-Rad Laboratories, Inc., Hercules, CA, USA). All serum samples were thawed and centrifuged at 10.000 × g at 4 °C for 10 min and further diluted (1:4) with sample diluent HB (Bio-Rad, Hercules, CA, USA) and 50 μL was loaded into 96-well plates. The multiplex analysis was performed according to the manufacture’s description. All wash steps were performed using the Bio-Plex Pro™ Wash Station (Bio-Rad, Hercules, CA, USA). An in-house spiked serum control was used to determine intra-assay and inter-assay percent of coefficient of variation (CV) that ranged from 0.4 to 7.8% and from 5.2 to 17.7%, respectively.

A representative selection of study participants and healthy control serum samples were screened with a 40 plex Bio-Plex Pro Human Chemokine panel (cat. no.: 171AK99MR2, Bio-Rad, Hercules, CA, USA), in addition to a separate measurement of TGF-β. This was done to select potentially relevant cytokines and to minimize analytical uncertainties by limiting the size of the final panel. Based on the screening with correlation analyses and literature search [27, 28, 50, 51], a custom-made 9 plex (Bio-Rad, Hercules, CA, USA), containing targets against macrophage colony–stimulating factor (GM-CSF), IL-1β, IL-4, IL-6, IL-8, interferon-γ-inducible protein 10 (IP-10), thymus- and activation-regulated chemokine (TARC), TNF, and epithelial-neutrophil activating peptide 78 (ENA-78), was used to analyze serum samples from 262 study participants and 100 healthy controls. One study sample was not brought to the laboratory by administrative failure.

### Statistical analysis

Descriptive statistics were presented as frequency and proportion for categorical data and median and range for continuous data. Comparison of groups was performed by the independent sample *t*-test, Mann–Whitney *U* test, and chi-square test as appropriate. Spearman’s rho was calculated to examine the correlation between cytokines, and between each cytokine and biochemical parameters, respectively, where *r* ≥ 0.6 was interpreted as moderate [52]. To investigate the associations between a panel of biomarkers in HNC survivors and controls, we performed a set of multinomial logistic regression analyses. The dependent variables had three categories (HNC survivors with and without the specific late effect (i.e., xerostomia, dysphagia, and CF) and controls). The cytokines served as covariates, while we adjusted for available variables in the controls, i.e., age and gender. To examine the associations between cytokines and late effects within the HNC survivors, we performed binary logistic regression analyses, where we also adjusted for comorbidity (yes/no), and further explorative analyses stratified by gender. As the cytokine values were highly skewed, outliers were truncated to five times the interquartile range to reduce the influence of extreme values. No multicollinearity between the independent variables was observed. Results are presented as odds ratios (OR) with 95% confidence intervals (CI). The statistical analyses were performed in SPSS version 26 and Stata version 16.

## Results

**Fig. 1:**
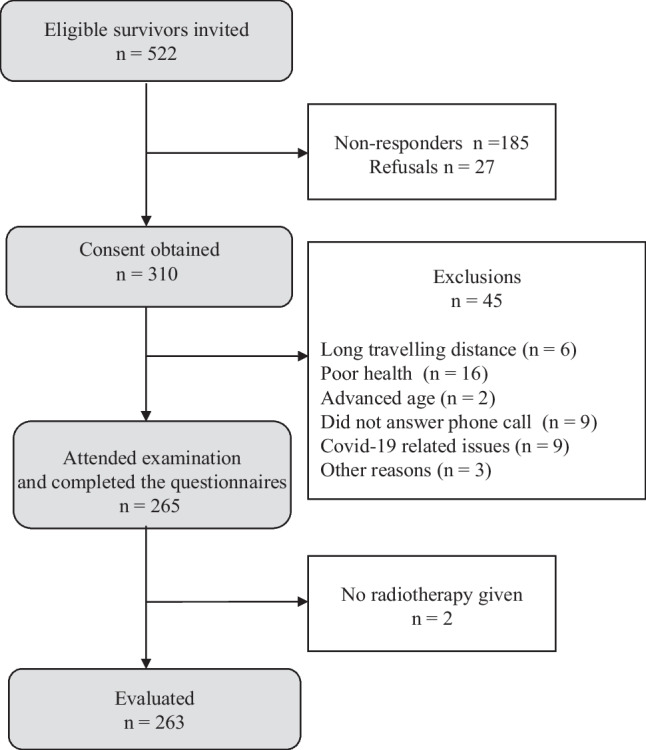
Flowchart of the study participant selection

Of the 522 HNC survivors identified from the hospital diagnosis register, 310 provided written informed consents. Forty-five of these later withdrew their consent, and two survivors were excluded since they had not received RT (Fig. 1). Of the 263 HNC survivors included, 148 also participated in the EORTC multi-center study [37]. In the survey, the 263 participants were younger (median age 65 years) than the survivors not included (median age 70 years), but there were no differences in gender or geographical distance to the hospital (Supplementary S2, Online Resource 1). The participants’ median age at diagnosis was 56 years, and the median time since treatment was 8.5 years (Table 1). There were 67% males, and 47% had one or more comorbidities. The majority had been treated for oropharyngeal cancer (52%) and had presented with stage III or IV disease (69%). Most of the participants had received RT as the primary modality (62%) or in a postoperative setting (33%). Concomitant chemotherapy was administered in 79% of cases of definitive RT, and only in 2% of the operated patients (Supplementary S3, Online Resource 1).

**Table 1:** Patient characteristics of long-term HNC survivors treated with radiotherapy

	All patients, *n* = 263	Xerostomia, *n* = 153	Dysphagia, *n* = 81	Chronic fatigue, *n* = 87
Age at diagnosis (years)				
Median (range)	56 (12–80)	56 (13–80)	61 (35–80)	56 (13–80)
Age at survey (years)				
Median (range)	65 (20–87)	65 (20–87)	69 (43–87)	63 (20–87)
Time from diagnosis to survey (years)				
Median (range)	8.5 (7–13)	8.2 (7–13)	8.7 (7–13)	8.5 (7–13)
Sex, *n* (%)				
Male	175 (67)	90 (41)	47 (58)	44 (51)
Female	88 (33)	63 (59)	34 (42)	43 (49)
Site of primary tumor, *n* (%)				
Oropharynx	137 (52)	78 (51)	40 (49)	45 (52)
Nasopharynx	7 (3)	6 (4)	5 (6)	5 (6)
Hypopharynx	4 (2)	3 (2)	3 (4)	3 (3)
Larynx	18 (7)	11 (7)	10 (12)	8 (9)
Oral cavity	45 (17)	34 (22)	13 (16)	11 (13)
Nose, sinuses	8 (3)	2 (1)	1 (1)	2 (2)
Unknown primary	12 (5)	6 (4)	3 (4)	2 (2)
Other *	32 (12)	13 (9)	6 (7)	11 (13)
Stage (UICC 7th edition), *n* (%)				
I	40 (15)	22 (14)	11 (14)	13 (15)
II	39 (15)	21 (14)	9 (11)	11 (13)
III	48 (18)	31 (20)	17 (21)	18 (21)
IV	135 (51)	78 (51)	43 (54)	44 (51)
Unknown	1 (1)	1 (1)	1 (1)	1 (1)
Histology, *n* (%)				
Squamous cell carcinoma	220 (84)	133 (87)	71 (88)	69 (79)
Salivary gland carcinomas	30 (11)	11 (7)	5 (6)	12 (14)
Undifferentiated carcinoma	8 (3)	7 (5)	4 (5)	5 (6)
Other**	5 (2)	2 (1)	1 (1)	1 (1)
HPV status				
No	33 (13)	20 (13)	10 (12)	10 (11)
Yes	74 (28)	41 (27)	21 (26)	26 (30)
Unknown	156 (59)	92 (60)	50 (62)	51 (59)
Cancer status, *n* (%)				
Recurrence free after primary treatment	220 (84)	125 (82)	66 (81)	68 (78)
Treated locoregional relapse ± second primary	26 (10)	17 (11)	8 (10)	9 (10)
Treated second primary only	17 (6)	11 (7)	7 (9)	10 (11)
Performance status, *n* (%)				
WHO 0	161 (61)	75 (49)	39 (48)	22 (25)
WHO 1	75 (29)	56 (37)	26 (32)	44 (51)
WHO ≥ 2	27 (10)	22 (14)	16 (20)	21 (24)
Comorbidities, *n* (%)				
Charlson Comorbidity Index (range 0–8)				
Score = 0	140 (53)	76 (50)	40 (49)	36 (41)
Score ≥ 1	123 (47)	77 (50)	41 (51)	51 (59)
-Cardiovascular disease	98 (47)¤			
-Rheumatic disease	34 (16)¤			
-Chronic pulmonary disease	25 (12)¤			
-Diabetes mellitus	22 (11)¤			
-Malignant disease	14 (7)¤			
-Other	16 (8)¤			
Living situation, *n* (%)				
Alone	64 (24)	40 (26)	24 (30)	25 (29)
Not alone	199 (76)	113 (74)	57 (70)	62 (71)
Education, *n* (%)				
< 10 years	45 (17)	28 (18)	20 (25)	17 (20)
= 10 years	32 (12)	19 (12)	11 (14)	8 (9)
> 10 years	186 (71)	106 (69)	50 (62)	62 (71)
Working situation, *n* (%)				
Employed/student	99 (38)	52 (34)	23 (28)	20 (23)
Unemployed	4 (2)	4 (3)	1 (1)	3 (3)
Unable to work	52 (19)	37 (24)	20 (25)	33 (38)
Retired	108 (41)	60 (39)	37 (46)	31 (36)
Smoking habits, *n* (%)				
Never	74 (28)	37 (24)	18 (22)	27 (31)
Former	146 (56)	83 (54)	46 (57)	42 (48)
Current	43 (16)	33 (22)	17 (21)	18 (21)
Pack years				
Median (range)	19 (0–112)	17 (0–112)	20 (1–60)	19 (0–98)
Drinking habits, *n* (%)				
Never	33 (13)	24 (16)	17 (21)	18 (21)
Monthly or less	54 (21)	30 (20)	19 (23)	18 (21)
2–4 times/month	58 (22)	36 (24)	17 (21)	16 (18)
2–3 times/week	88 (33)	45 (29)	21 (26)	27 (31)
4–5 times/week	30 (11)	18 (12)	7 (9)	8 (9)
Feeding tube, *n* (%)	9 (3)	7 (5)	9 (11)	7 (8)

^*^Includes primary site in salivary glands and lip

^**^Other; melanoma *n* = 1, sarcoma *n* = 1, adenocarcinoma/carcinoma with endocrine differentiation *n* = 3

¤% of 209 events, more than one comorbidity possible

Pack years: (number of cigarettes × number of years)/20)

Of the 263 HNC survivors examined, 58% had xerostomia, 31% had dysphagia, and 33% had CF and the characteristics for each subgroup are displayed in Table 1. Females reported more xerostomia (72% versus 52%), dysphagia (39% versus 27%), and CF (49% versus 25%) compared to males. All study participants completed the PROMs, and there were no missing values for these symptoms.

Biochemical parameters, radiation dose, use of chemotherapy, BMI, and smoking status were similar in survivors reporting late effects and those who did not (Supplementary S4, Online Resource 1).

**Fig. 2:**
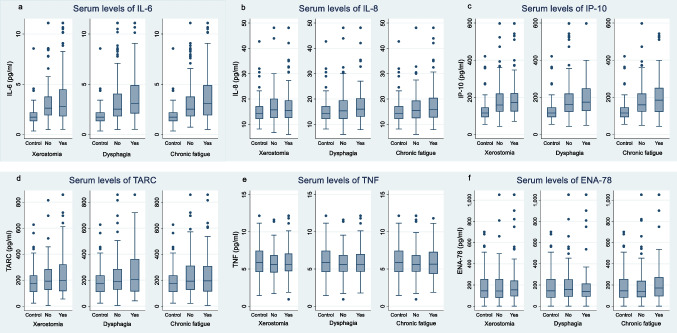
Cytokine levels in head and neck cancer survivors with (yes) and without (no) xerostomia, dysphagia, and chronic fatigue more than 5 years post-treatment. The cytokine levels in healthy controls are also shown. Each plot shows the median values with whiskers and outliers of **a** IL-6, **b** IL-8, **c** IP-10, **d** TARC, **e** TNF, and **f** ENA-78

In both the study group and in the control group, levels of GM-CSF, IL-1β, and IL-4 were either below detection or reported with high uncertainty (stipulated values), and were therefore omitted. The remaining six cytokines IL-6, IL-8, IP-10, TARC, TNF, and ENA-78 were included in the final analyses. There were higher serum levels of IL-6, IL-8, and IP-10 in study participants compared to controls, while no differences were observed for TARC, TNF, and ENA-78 (Fig. 2a-f; Supplementary S5, Online Resource 1). When mutually adjusting for all cytokines as well as age and gender, the multinomial regression analyses showed positive associations for IL-6 and IP-10, and negative associations for TNF in HNC survivors both with and without late effects compared to controls (Table 2). An increase in pg/mL:5 in IL-6 was associated with an OR of 41–74 and an increase in pg/mL:50 in IP-10 was associated with an OR of 1.82–1.97 for HNC survivors compared to controls (all *p* values < 0.001).

**Table 2:** Multinominal logistic regression analyses of factors associated with late effects in long-term HNC survivors (*n* = 262) compared to age- and gender-matched healthy controls (*n* = 100)

	Xerostomia		Dysphagia		Chronic fatigue	
	Yes	No	Yes	No	Yes	No
	OR (95% CI)	OR (95% CI)	OR (95% CI)	OR (95% CI)	OR (95% CI)	OR (95% CI)
Age at survey (years)	1.01 (0.98–1.05)	1.01 (0.98–1.05)	**1.06 (1.01–1.11)**	1.00 (0.97–1.03)	1.00 (0.96–1.03)	1.02 (0.97–1.06)
Gender (females vs. males)	0.56 (0.29–1.10)	1.37 (0.66–2.82)	0.50 (0.24–1.07)	0.97 (0.50–1.87)	**0.40 (0.20–0.83)**	1.18 (0.61–2.29)
IL-6 (pg/mL:5)	58 (12–267)**	42 (8.83–196)**	57 (12–279)**	46 (10–211)**	74 (15–361)**	41 (8.88–188)**
IL-8 (pg/mL:5)	0.92 (0.68–1.25)	1.01 (0.75–1.37)	0.98 (0.71–1.37)	0.96 (0.72–1.27)	1.04 (0.75–1.43)	0.93 (0.69–1.24)
IP-10 (pg/mL:50)	1.94 (1.46–2.58)**	1.92 (1.44–2.56)**	1.82 (1.35–2.45)**	1.97 (1.48–2.61)**	1.93 (1.44–2.59)**	1.93 (1.46–2.56)**
TARC (pg/mL:50)	1.07 (0.94–1.21)	1.01 (0.89–1.16)	1.08 (0.94–1.24)	1.03 (0.91–1.16)	1.02 (0.89–1.17)	1.05 (0.93–1.19)
TNF (pg/mL)	0.78 (0.66–0.93)*	0.74 (0.62–0.88)*	**0.78 (0.64–0.95)**	0.75 (0.64–0.89)*	0.76 (0.63–0.92)*	0.76 (0.64–0.90)*
ENA-78 (pg/mL:50)	1.00 (0.90–1.10)	1.00 (0.90–1.12)	0.99 (0.89–1.11)	1.00 (0.91–1.11)	0.99 (0.89–1.11)	1.00 (0.91–1.11)

*OR*, odds ratio; *CI*, confidence interval; *IL*, interleukin; *IP-10*, interferon-γ-inducible protein 10; *TARC*, thymus- and activation-regulated chemokine; *TNF*, tumor necrosis factor; *ENA-78*, epithelial-neutrophil activating peptide-78

The healthy control group served as the reference category of the dependent variable

Significant associations given in bold letters: *p* < 0.05, *p < 0.01, **p < 0.001

Within HNC survivors, we did not exhibit any associations between cytokine levels and the late effects xerostomia, dysphagia, and CF (Table 3). However, females had lower OR of dysphagia and CF compared to males. Explorative analyses of associations between cytokine levels and late effects stratified by gender yield similar results (Supplementary S6, Online Resource 1).

**Table 3:** Multivariable logistic regression analyses of factors associated with late effects in long-term HNC survivors (*n* = 262)

	Xerostomia		Dysphagia		Chronic fatigue	
	OR	95% CI	OR	95% CI	OR	95% CI
Age at survey (years)	1.05*	1.02–1.08*	0.99	0.97–1.02	**0.97**	**0.94–1.00**
Gender (females vs. males)	0.58	0.34–1.01	0.40 *	0.22–0.71*	0.31**	0.17–0.57**
Comorbidity (no/yes)	1.25	0.74–2.11	1.50	0.87–2.57	2.74*	1.49–5.03*
IL-6 (pg/mL:5)	1.54	0.96–2.49	1.25	0.69–2.28	1.43	0.78–2.62
IL-8 (pg/mL:5)	1.19	0.99–1.43	0.89	0.70–1.13	1.10	0.85–1.41
IP-10 (pg/mL:50)	1.03	0.92–1.15	1.02	0.90–1.16	0.99	0.86–1.13
TARC (pg/mL:50)	1.07	0.98–1.16	1.05	0.96–1.16	0.98	0.89–1.08
TNF (pg/mL)	1.06	0.93–1.21	1.05	0.91–1.23	0.97	0.83–1.15
ENA-78 (pg/mL:50)	1.02	0.95–1.10	0.99	0.91–1.08	1.00	0.91–1.10

*OR*, odds ratio; *CI*, confidence interval; *IL*, interleukin; *IP-10*, interferon-γ-inducible protein *10*; *TARC*, thymus- and activation-regulated chemokine; *TNF*, tumor necrosis factor; *ENA-78*, epithelial-neutrophil activating peptide-78

Significant associations given in bold letters:* p* < 0.05, *p < 0.01, **p < 0.001

## Discussion

We found that more than half of the survivors had persisting problems with xerostomia and that approximately one-third had dysphagia and CF more than 5 years after RT. In order to get a better understanding of the biological mechanisms involved, we explored potential links between late effects and biomarkers. We did not find any significant associations between a selection of pro-inflammatory cytokines and xerostomia, dysphagia, or CF among HNC survivors. Although these results were negative, HNC survivors had higher levels of some cytokines compared to healthy controls. This could indicate persistent systemic changes in the survivors.

The high prevalence of xerostomia in this study verifies that this symptom is a long-term problem for many HNC survivors [53]. Our result is in line with prior reports on xerostomia being a significant problem in the first years following treatment and increases the understanding of the irreversibility of this condition [19, 54, 55]. This should encourage health care professionals to inform HNC survivors that xerostomia commonly persists, and provide them with advice including how to prevent oral and dental complications such as candida infection and caries.

Higher prevalence of dysphagia was found in this study (31%) compared to the previously reported prevalence of 14% at 2 years [56]. This may be due to development of soft tissue fibrosis, which tend to progress over time [31, 57]. The finding of this study is of great importance as literature on long-term patient-reported dysphagia is scarce. One small study of 39 HNC survivors found that 26% had persistent patient-reported dysphagia at 5 years, while five of the 21 individuals (24%) who underwent assessment of swallowing function by videofluoroscopy had impaired function [58].

Until now, data on CF in HNC survivors have been limited, but this study showed that as many as one-third of the survivors suffered from this condition. While a previous report of a more selected HNC population described lower levels [59], the proportion of HNC survivors affected by CF in this study was at the same high level as for survivors treated for breast cancer and lymphoma [23, 26, 36]. This added information suggests that a considerable number of HNC survivors may require assistance and guidance in handling CF, and efforts should be made to accommodate these needs.

The finding that late effects were more prevalent in females than in males is consistent with other reports [60]. In the Norwegian reference population scores for the EORTC QLQ-C30 [61], women reported more problems with functioning, more symptoms, and lower overall quality of life compared to men, across different age groups. This argues for gender differences being a general issue and not specific to HNC survivors. Such gender variety may be genetic, biological, behavioral, or a combination [62–64].

We found higher levels of IL-6 and IP-10 in HNC survivors, independent of reported late effects, compared to blood donor controls. This is an interesting observation as the explanation is not known, and similar findings have not been reported previously. Both these cytokines are associated with tumor progression and poor survival in cancer patients [65–68]. IL-6 is a multifunctional protein and has both pro- and inflammatory properties. It is an important component of the immune system, as well as of the cancer microenvironment and in other biological processes such as aging, autoimmune processes, and hematopoiesis [65, 69]. IP-10 is a chemokine which, through binding to its receptor chemokine (C-X-C motif) receptor 3 (CXCR3), attracts immune cells to inflammatory sites. It is involved in several biological processes, especially related to autoimmune diseases, and is linked to lymphocyte infiltration at tumor cancer sites [67, 70, 71]. How this might be connected to being a HNC survivor is unknown, but may be related to an ongoing inflammatory process and as such our result may be hypothesis generating for future studies. We were surprised to find that the HNC survivors did not have higher levels of TNF and TGF-β than the controls, as these cytokines have been linked to fibrotic processes following irradiation [4, 32–34].

We did not observe any associations between levels of IL-6, IL-8, IP-10, TARC, TNF, and ENA-78 in the HNC survivors who reported xerostomia, dysphagia, and CF scores above thresholds, and those who did not. Because the subgroups did not exhibit differences in tobacco smoking and BMI, these potential confounders were not included in the final models. Documentation of pro-inflammatory cytokines in HNC survivors is scarce and predominantly detected in saliva in the early years after treatment [29, 30, 72]. In one study, increased serum levels of IL-1β, IL-6, and IL-10 were detected 7 weeks after treatment initiation with return to pre-treatment levels at 3 months, but IL-1β and IL-10 were slightly rising again 1 year after treatment completion. However, the observed cytokine levels were not explored in connection to treatment-related side effects [3]. Persistent inflammation as an etiological factor has primarily been proposed and investigated for cancer-related fatigue [26]. One of the hypotheses is that activation of peripheral pro-inflammatory cytokine networks transmits signals to the brain, which promote sickness behavior [27, 36]. Although several publications have indicated an association between fatigue and systemic cytokines such as IL-1β, IL-2, IL-6, IP-10, and TNF in cancer patients, the results are conflicting. Retrospective study design, small cohorts, and incomparable cytokine quantification methods across studies limit the significance of the results [28, 51, 67, 73]. Ongoing acute infections, autoimmune diseases, aging, smoking, and physical activity can potentially give elevated levels of pro-inflammatory cytokines [65, 74, 75], but there is limited knowledge in this field. We cannot rule out high levels of cytokines in the HNC survivors’ blood samples due to other causes. In addition, cytokines have short half-life, and they can degrade during sampling handling which may give false low measurements, or release from cells during storage can lead to false elevated levels [76].

A strength of this study was the inclusion of an unselected sample of HNC patients treated at OUH during a 5-year period. The participants matched survivors who declined to participate by gender and travel distance to the hospital, but not by age. Still, the age distribution of the participants was broad, ranging from 20 to 87 years. Another strength was the involvement of user representatives from the start, who pointed out the insufficient knowledge of late effects after completion of the 5-year follow-up. They emphasized that many HNC survivors are struggling alone without adequate support and lack relevant information and coping strategies. By taking the aspects of user representatives into account, we believe that the relevance of this research was enhanced, which is also in accordance with the National Cancer Strategy of Norway 2018–2022 [77]. Using PROMs is an advantage compared to observer-reported outcomes, as observers tend to underrate the patients’ experiences and introduce inter-rater variability [9, 10]. Xerostomia, dysphagia, and CF are subjective experiences and best reported by patients themselves [11, 78]. The 100% compliance and no missing values in the completed PROMs strengthen the results of this study and underline the importance of having dedicated study personnel to monitor data collection closely. Experienced personnel carefully handled blood sampling, storage, and cytokine analyses according to procedures, which minimized the uncertainties. All of our study samples were collected in the morning, to eliminate discrepancy as several cytokines have a diurnal variation [74]. By recruiting healthy blood donor controls, we were able to obtain a basis for comparison of cytokine levels against the study samples as cut-offs have not yet been established for pro-inflammatory cytokines, partly because there is a lack of standardization of analyzing methods [76].

The cross-sectional design was a limitation of this study as the pre-treatment status of the participants was not available and it does not account for possible pre-existing xerostomia, dysphagia, and CF. Future strategies would be to use prospectively registered data in the evaluation of late effects. In addition, the choice of the EORTC QLQ-H&N35 single-item scale to measure dry mouth might be questioned, as single items are known to be less robust than multi-item scales [78]. Hence, other questionnaires such as Xerostomia Questionnaire, Groningen Radiotherapy-Induced Xerostomia Scale, and Summated Xerostomia Inventory-Dutch version could have provided more robust results [79–81]. In 2020, the final year of our study, the SARS-CoV-2 pandemic might have imposed a selection bias by causing delays and withdrawals, primarily affecting individuals > 70 years. Younger patients were more likely to have received comprehensive and/or multimodal therapy with increased risk of late effects, which in turn would motivate them to take part in this study.

## Conclusions

This study provided new knowledge on late effects in HNC survivors more than 5 years after receiving IMRT and chemoradiotherapy introduced 10–15 years ago. This can serve as grounds for informing patients with HNC about risks of these late effects. Improved self-management and coping may reduce the need for health services. The results may contribute to increased understanding of HNC survivors’ long-term challenges among clinicians, dental personnel, speech pathologists, nutritionists, physiotherapists, psychologists, and other social actors, e.g., the Norwegian Labor and Welfare Administration (NAV) and social workers. Further efforts should be made to better comprehend the biological mechanisms, for instance by collecting biomaterial in a prospective longitudinal study. This is time-consuming and demanding, but it will be worth the endeavors if preventive measures and better customized management of HNC patients can be achieved. There is also a need for more interventional studies, which aim to improve or treat the late effects.

## Supplementary information

Below is the link to the electronic supplementary material.

Supplementary file1 (PDF 89 KB)

## Data Availability

The datasets generated and/or analyzed during the current study are available from the corresponding author on reasonable request.
