# Supplementary material for: Associations between patient-reported late effects and systemic cytokines in long-term survivors of head and neck cancer treated with radiotherapy
Source: J Cancer Surviv. 2022 Nov 9;17(4):1082–93. doi: 10.1007/s11764-022-01273-1 (PMC9643919; doi:10.1007/s11764-022-01273-1)
Supplement: Supplementary file 1 — Supplementary file1 (PDF 89 KB) [file 11764_2022_1273_MOESM1_ESM.pdf]

Supplementary material

Title: Associations between patient-reported late effects and systemic cytokines in long-term survivors of head and neck cancer treated with radiotherapy

Authors

Huynh TTM (1,2), Aass HCD (3), Falk RS (4), Astrup GL (2), Helland Å (1,2), Bjøro T (1,3), Bjordal K (1,4), Dale E (2), Hellebust TP (5,6), Herlofson BB (7,8), Malinen E (5,6), Kiserud CE (2), Osnes T (1,8), Amdal CD (2,4)

Affiliations

(1) Faculty of Medicine, University of Oslo, Oslo, Norway

(2) Department of Oncology, Oslo University Hospital, Oslo, Norway

(3) Department of Medical Biochemistry, Oslo University Hospital, Oslo, Norway

(4) Research support services, Oslo University Hospital, Oslo, Norway

(5) Department of Physics, University of Oslo, Oslo, Norway

(6) Department of Medical Physics, Oslo University Hospital, Oslo, Norway

(7) Faculty of Dentistry, University of Oslo, Oslo, Norway

(8) Department of Otorhinolaryngology, Head and Neck Surgery, Oslo University Hospital, Oslo, Norway

Corresponding author

Thuy-Tien Maria Huynh

Department of oncology, Oslo University Hospital, Oslo, Norway.

Postal address: Oslo University Hospital, Post Box 4950 Nydalen, NO-0424 Oslo, Norway.

E-mail: thuhuy@ous-hf.no. Phone: +47 9289664.

Supplementary S1. Serum preparation, biochemical analyses, and blood donor control selection

For cytokine analyses, blood was drawn into either 4 mL or 8 mL gel free serum tubes (Vacuette, Greiner, Austria) and kept at room temperature between 30-60 minutes prior to 1000xg centrifugation for 15 min at 4ºC. The serum samples were subsequently stored at -80ºC and thawed before use.

Biochemical analyses in study participants were conducted according to hospital's procedures.

We obtained approval from The Regional Committees for Medical and Health Research Ethics before inclusion of healthy controls. They were recruited from the Blood Bank, OUH, and provided written informed consents before inclusion. The healthy controls were frequency matched with the study population by age and gender, thus 30% females and 70% males were selected. This resulted in a study participant-to-control-ratio of 2.5 for males and 3 for females, respectively. The upper age limit for blood donators is 70 years. Hence, we were not able to conduct individual age-matching, but we used frequency matching to achieve a best possible balance in the age distribution between the study population and the healthy controls.

Supplementary S2. Comparison of eligible study candidates

	Included	Not included*
N	n=263	n=259
Gender, n (%)		
Male	175 (67)	166 (64)
Female	88 (33)	92 (36)
Unknown		1
Age in years, median (range)	65 (21-87)	70 (16-96)
Geographical distance between residence and OUH, n (%)		
< 1 hour	111 (42)	101 (39)
1-2 hours	96 (37)	103 (40)
>2 hours	56 (22)	54 (21)
Unknown		1

*Comprises non-responders, individuals who actively declined, individuals who withdrew their consent, and candidates excluded due to other causes

Supplementary S3. Treatment characteristics of long-term HNC survivors (n=263)

	**Primary irradiation**<br>n = 164	**Postoperative irradiation**<br>n = 86	**Radiation at relapse**<br>n = 13
**Modality, n (%)**			
IMRT	115 (70)	22 (26)	5 (38)
3DCRT/direct field determination	49 (30)	64 (74)	8 (62)
**Radiation dose (Gy)**			
Mean (range)	70 (51-74)	59 (51-74)	60 (50-72)
**Neck irradiation, n (%)**			
Bilateral	130 (79)	37 (43)	9 (69)
Unilateral	32 (20)	30 (35)	3 (23)
No	2 (1)	19 (22)	1 (8)
**Concomitant chemotherapy, n (%)**			
Yes	130 (79)	2 (2)	1 (8)
No	34 (21)	84 (98)	12 (92)
**Targeted therapy, n (%)**			
Yes	22 (13)	0 (0)	0 (0)
No	142 (87)	86 (100)	13 (100)
**Nimorazole, n (%)**			
Yes	152 (93)	2 (2)	1 (8)
< 15 days	15 (8)	0 (0)	0 (0)
≥ 15 days	137 (84)	2 (2)	1 (8)
No	12 (7)	84 (98)	12 (92)

Supplementary S4. Frequency (proportion) and median (range) of biochemical parameters, radiation dose, use of chemotherapy, BMI, and smoking status in HNC survivors by presence of late effects

	Xerostomia		Dysphagia		Chronic fatigue	
	Yes (n=153)	No (n=110)	Yes (n=81)	No (n=182)	Yes (n=87)	No (n=176)
**Age at survey (years)**<br>median<br>(range)	65<br>(20-87)	66<br>(23-83)	69<br>(43-87)	64<br>(20-84)	63<br>(20-87)	66<br>(23-84)
**Body mass index (kg/m2)**<br>median<br>(range)	25.9<br>(16.7-47.0)	26.2<br>(18.2-43.0)	25.0<br>(16.7-36.8)	26.5<br>(17.1-47.0)	26.8<br>(18.1-47.0)	26.0<br>(16.7-38.1)
**Cigarette smoking (pack years)**<br>median<br>(range)	10<br>(0-112)	10<br>(0-50)	13<br>(0-60)	7<br>(0-112)	9<br>(0-98)	10<br>(0-112)
**CRP (mg/L)**<br>median<br>(range)	1.4<br>(<0.6-108)	1.2<br>(<0.6-37)	1.6<br>(<0.6-108)	1.2<br>(<0.6-37)	1.5<br>(<0.6-108)	1.3<br>(<0.6-37)
**Hb (g/dL)**<br>median<br>(range)	14.2<br>(10.6-18.0)	14.4<br>(10.8-17.1)	13.8<br>(10.6-16.4)	14.5<br>(10.8-18.0)	13.7<br>(10.6-16.7)	14.4<br>(11.2-18.0)
**TSH (x10E-3 IU/L)**<br>median<br>(range)	2.9<br>(<0.01-16)	2.7<br>(<0.01-15)	3.3<br>(<0.01-16)	2.7<br>(0.02-11)	2.8<br>(0.02-16)	2.8<br>(<0.01-13)
**FT4 (pmol/L)**<br>median<br>(range)	15<br>(10-26)	15<br>(11-23)	15<br>(10-26)	15<br>(10-24)	15<br>(11-26)	14<br>(10-24)
**PTH (pmol/L)**<br>median<br>(range)	4.7<br>(1.3-14.2)	4.5<br>(2.1-10.3)	4.7<br>(1.3-10.5)	4.6<br>(2.3-14.2)	4.6<br>(2.6-13.4)	4.6<br>(1.3-14.2)
**Calcium (mmol/L)**<br>median<br>(range)	2.4<br>(2.2-2.6)	2.4<br>(2.1-2.6)	2.4<br>(2.2-2.6)	2.4<br>(2.1-2.6)	2.4<br>(2.2-2.6)	2.4<br>(2.1-2.6)
**Sodium (mmol/L)**<br>median<br>(range)	141<br>(134-145)	141<br>(135-145)	141<br>(134-145)	141<br>(135-145)	141<br>(134-144)	141<br>(135-145)
**Potassium (mmol/L)**<br>median<br>(range)	4.1<br>(3.3-5.0)	4.1<br>(3.3-4.6)	4.0<br>(3.3-5.0)	4.1<br>(3.3-4.9)	4.1<br>(3.3-5.0)	4.1<br>(3.3-4.9)
**Vitamin B12 (pmol/L)**<br>Median<br>(range)	346<br>(137-2950)	348<br>(151-1143)	342<br>(137-2950)	350<br>(151-2330)	359<br>(141-1143)	342<br>(137-2950)
**Vitamin D (25-OH nmol/L)**<br>median<br>(range)	69<br>(20-152)	74<br>(29-163)	73<br>(30-132)	71<br>(20-163)	69<br>(28-155)	72<br>(20-163)
**Radiation dose (Gy)**<br>Median (range)	70 (50-74)	70 (50-72)	70 (50-70)	70 (50-74)	70 (50-74)	70 (50-72)
**No neck radiation**<br>n (%)	4 (3)	18 (16)	2 (2)	20 (11)	5 (6)	17 (10)
**Unilateral neck radiation**<br>n (%)	35 (23)	30 (27)	15 (19)	50 (27)	21 (24)	44 (25)
**Bilateral neck radiation**<br>n (%)	114 (75)	62 (56)	64 (79)	112 (62)	61 (70)	115 (65)
**Chemotherapy**<br>n (%)	79 (52)	54 (49)	43 (53)	90 (49)	46 (53)	87 (49)
**Physical activity > 30 min daily**<br>n (%)	131 (86)	102 (93)	70 (88)	163 (90)	74 (85)	159 (90)
**Hours of inactivity daily**<br>Median (range)	6 (2-15)	5 (1-18)	6 (1-15)	6 (1-18)	6 (2-15)	5 (1-18)

Supplementary S5. Cytokine and CRP levels in study participants, total and pr. late effect, and in controls. Supplementary to Figure 2

**Cytokine**	**Controls** n=100	**All study participants** n=262	**Xerostomia** n=152	**Dysphagia** n=80	**Chronic fatigue** n=87
IL-6	2 (0-9)	**3 (1-11)****	**3 (1-11)****	**3 (1-11)****	**3 (1-11)****
IL-8	14 (8-43)	**16(6-48)**	**15 (6-48)**	**16 (8-48)***	**16 (8-48)**
IP-10	116 (54-421)	**169 (44-598)****	**173 (71-598)****	**174 (50-598)****	**185 (44-543)****
TARC	175 (24-627)	195 (6-857)	196 (55-857)	204 (42-857)	195 (6-857)
TNF	6 (2-12)	6 (1-12)	6 (1- 12)	6 (2-12)	6 (2-12)
ENA-78	146 (0-701)	152 (0-1051)	155 (1-1051)	139 (1-1051)	172 (0-1051)
CRP¤	-	1 (1-108)	1 (1-108)	2 (1-108)	2 (1-108)

IL: Interleukin, IP-10: Interferon-γ-inducible protein 10, TARC: Thymus- and activation-regulated chemokine, TNF: Tumor necrosis factor, ENA-78: Epithelial-neutrophil activating peptide-78, CRP: C-reactive protein.

Values given as pg/ml for cytokines and mg/L for CRP, median (range). Detection limit 0.6 mg/L for CRP.

Mann Whitney U tests, where significant associations compared to controls are given in bold letters

*p < 0.01; **p < 0.001

¤CRP was only measured in the study population, consequently not compared to controls.

Supplementary S6. Logistic regression analyses, late effects in study participants, stratified by gender

	**Male** (n = 175)		**Female** (n = 88)	
	OR	95% CI	OR	95% CI
**Xerostomia**				
Age at survey (years)	0.99	0.96-1.02	1.01	0.96-1.06
Comorbidity	1.32	0.71-2.44	2.01	0.66-6.14
IL-6 (pg/ml:5)	0.87	0.48-1.58	4.52	0.80-25.57
IP-10 (pg/ml:50)	1.04	0.90-1.19	0.92	0.71-1.20
**Dysphagia**				
Age at survey (years)	**1.06***	**1.01-1.12***	1.05	1.00-1.10
Comorbidity	0.73	0.36-1.49	1.59	0.61-4.17
IL-6 (pg/ml:5)	1.20	0.62-2.32	1.71	0.69-4.28
IP-10 (pg/ml:50)	0.97	0.82-1.14	0.89	0.72-1.11
**Chronic fatigue**				
Age at survey (years)	0.97	0.93-1.01	0.99	0.94-1.04
Comorbidity	**2.33***	**1.07-5.06***	2.79	0.99-7.84
BMI	1.01	0.93-1.10	1.08	0.98-1.20
IL-6 (pg/ml:5)	1.35	0.71-2.56	2.35	0.82-6.69
IP-10 (pg/ml:50)	1.04	0.88-1.22	0.83	0.65-1.05

OR: Odds ratio, CI: Confidence interval IL-6: Interleukin-6, IP-10: Interferon-γ-inducible protein 10, BMI: Body mass index. Significant associations given in bold letters.

*$p < 0.05$
